# Heatwaves and health system resilience in Europe

**DOI:** 10.1016/j.eclinm.2026.104168

**Published:** 2026-08-24

**Authors:** eClinicalMedicine

As Europe, the fastest-warming continent, sustains recent record-breaking heatwaves that began in June, 2026, extreme temperatures are continuing to cause heat-related illness and excess deaths. The heatwaves are testing the resilience of health systems and intensifying the need for adaptation of health systems to a warming climate.

Extreme heat places considerable strain on human health and is associated with a significant increase in mortality. Heat-related illnesses disproportionately affect vulnerable populations, including older adults, children, pregnant women, outdoor workers, people experiencing homelessness, and individuals living with chronic diseases. According to the latest *Lancet Countdown on health and climate change* yearly report, heat-related mortality increased in 99·6% of monitored European regions between 2015 and 2024, averaging 52 additional deaths per million inhabitants each year compared with 1991–2000. Thus far, the 2026 heatwaves have been particularly hazardous because persistently elevated temperatures have extended exposure periods, increasing the risk of heat-related illness even among healthy populations.

This summer’s heat crisis has also highlighted the vulnerability of health systems, many of which are struggling to cope with the surge in patients presenting with heat-related illnesses such as dehydration, sunburn, heat exhaustion and heat stroke, and cardiovascular events. Extreme heat threatens the reliability of crucial health-care infrastructure, including electricity supplies for medical equipment, cooling and technology systems, medication storage and transport, as well as workforce productivity. When any of these components fail, the quality and continuity of patient care can be compromised. Additionally, the persistence of the weather effect continues to increase morbidity and hospital admission for worsening cardiovascular, respiratory, mental, and neurological conditions even after the temperature peak has passed. Despite growing recognition of these risks, the implementation of heat-resilient health-care systems remains inconsistent across Europe. Evidence-based strategies for surge planning, infrastructure resilience, and health-system adaptation are yet to be widely adopted, even in some of Europe’s most advanced health-care settings.

Multiple barriers prevent health systems responding to heatwaves effectively. Although weather forecasting enables increasingly accurate forewarning of temperature rises, health-care services are often unable to prepare at the speed required. Hospitals cannot readily increase cooling capacity, expand staffing levels, or reconfigure services in response to heat episodes forecasted several days in advance. At the same time, the heterogeneous presentation of heat-related illnesses across different patient groups and clinical specialties limits the feasibility of a universal preparedness protocol by medical departments across regions. Forecasts cannot guarantee that vulnerable populations will receive or act upon protective advice in time. Yet the effects of heatwaves on community-based health-care services (eg, primary care, long-term care, and nursing homes), which play a crucial role in supporting these groups, remain largely unquantified. Tools such as European Heat Health System and WHO’s Heat-Health Action Plans (updated in June, 2026) are available, offering guidance on how to monitor those most at risk and prepare health systems; however, little is known about the extent to which recommended adaptation measures are implemented or translated into effective public health action and operational responses. Efforts have largely focused on environmental and municipal planning, yet health-care systems often bear the immediate consequences of extreme heat.

Despite these challenges, both immediate and long-term measures exist that health systems can adopt to protect populations during heat. In Romania, dedicated hospital units for patients with heatstroke have helped to streamline clinical management, while, in the UK, colour-coded heat alerts linked directly to recommended actions for health-care practitioners have improved preparedness and response. WHO/Europe is promoting the use of the Hospital Safety Index across countries to assess hospitals’ ability to remain operational during emergencies. However, future preparedness will require more integrated approaches. A 2025 systematic review suggests that community-based support can complement health-system preparedness, with heat adaption behavioural interventions helping to protect older adults, socially isolated individuals, and others at elevated risk during heatwaves. However, most interventions remain fragmented and lack a comprehensive guiding framework. Heat-health warning systems in European countries should evolve beyond meteorological forecasting alone by incorporating climate, epidemiological, and health-care data to better predict individual and population-level health risks in different health sectors across regions and strengthen responses to recurrent heat extremes. Emerging technologies, including artificial intelligence, could further strengthen health-care responses by linking heat-hazard forecasts with health outcome predictions, resource allocation, and surge-capacity planning. Equally important is the effective communication of warnings within and across countries, supported by community engagement to reach vulnerable populations and stronger collaboration between the health sector, policy makers, and national and non-governmental organisations. Frameworks from the Global Heat Health Information Network and other WHO-World Meteorological Organization joint programmes offer practical guidance on heat risk governance and international cooperation. Finally, adaptation cannot be achieved without sustained investment. Building heat-resilient health systems requires explicit financing strategies that support infrastructure upgrades, workforce preparedness, surveillance systems, emergency response capacity, and long-term climate adaptation.

Greater emphasis must be placed on strengthening health-care system resilience and implementing existing frameworks in the face of extreme heat, which is rapidly becoming the new normal in Europe. This effort should encompass infrastructure adaptation, workforce protection, and coordinated surge-capacity planning. Although effective tools and frameworks already exist, their implementation across the health sector remains insufficient. Bridging this gap through coordinated action across governments, health systems, and other sectors will be essential if Europe is to avoid repeating the health emergencies witnessed during recent heatwaves.

